# B-Mode ultrasound imaging in diagnosing carpal tunnel syndrome: an auxiliary diagnostic tool for hand surgeons

**DOI:** 10.3389/fneur.2024.1325464

**Published:** 2024-01-29

**Authors:** Qiang Chen, Xiaodi Zou, Yanting Xia, Yingnan Hu, Congxian Chen, Ping Zheng

**Affiliations:** ^1^Center for Plastics & Reconstructive Surgery, Department of Hand & Reconstructive Surgery, Zhejiang Provincial People’s Hospital, Affiliated People’s Hospital, Hangzhou Medical College, Hangzhou, Zhejiang, China; ^2^Department of Orthopedics, The Second Affiliated Hospital of Zhejiang Chinese Medical University, Hangzhou, Zhejiang Province, China; ^3^Department of Gastrointestinal Surgery, The Second Affiliated Hospital of Zhejiang Chinese Medical University, Hangzhou, Zhejiang Province, China; ^4^Cancer Center, Department of Ultrasound Medicine, Zhejiang Provincial People's Hospital, Affiliated People's Hospital, Hangzhou Medical College, Hangzhou, Zhejiang Province, China; ^5^Department of Plastics, Tiantai People’s Hospital of Zhejiang Province (Tiantai Branch of Zhejiang Provincial People’s Hospital), Hangzhou Medical College, Taizhou, Zhejiang, China

**Keywords:** B-ultrasound diagnosis, flattening ratio, carpal tunnel syndrome (CTS), carpal canal volume, surgery

## Abstract

**Objective:**

The purpose of this article is to explore the effectiveness of B-Mode ultrasound as an auxiliary diagnostic tool for carpal tunnel syndrome (CTS). It aims to demonstrate the advantages of B-Mode ultrasound, including its non-invasive nature and its ability to provide real-time imaging, in localizing nerve compression and predicting postoperative outcomes.

**Methods:**

The study included 40 patients who were subjected to preoperative B-ultrasonography. The approach focused on evaluating the consistency of B-Mode ultrasound results with intraoperative findings. It also assessed the importance of employing standardized imaging techniques and emphasized the need for cooperation between hand surgeons and sonographers for accurate diagnosis.

**Results:**

B-Mode ultrasound findings in the study were consistent with intraoperative observations, indicating its reliability. Additionally, B-Mode ultrasound was able to identify other anatomical abnormalities within the carpal canal that may contribute to CTS symptoms, such as persistent median arteries, median nerve bifurcation, and space-occupying lesions like cysts and tumors.

**Conclusion:**

The article concludes that B-Mode ultrasound should be considered a valuable supplementary diagnostic tool for CTS, particularly in instances where clinical signs and electrophysiological studies do not offer clear results. However, it should not replace established diagnostic methods for CTS.

## Introduction

Carpal tunnel syndrome (CTS) is the most common disease that compresses the nerves of the upper limb and causes neuropathy of the limb. Common causes of CTS include thickening of the transverse carpal ligament or a reduction in carpal tunnel volume. Surgical incision of the transverse carpal ligament and median nerve decompression is the most common surgical procedure (1, 2). In recent years, with the improvement of B-Mode ultrasound equipment and doctors’ technology, B-Mode ultrasound has emerged as a valuable tool for evaluating CTS, offering unique advantages. As a convenient examination method, B-Mode ultrasound can be performed immediately on the same day in the outpatient department, is non-invasive, and is cost-effective, making it well-received by patients. It can measure the carpal tunnel volume, track changes in the median nerve’s circumference, assess the stenosis or enlargement of the median nerve, pinpoint the location of nerve entrapment, and facilitate the statistical analysis of nerve changes before and after surgery. Compared to other examinations, B-Mode ultrasound has a prominent advantage in distinguishing between the acute and chronic phases of CTS during sonographic examinations (3). During the acute phase, there can be extravasation of fluids within the endoneurial compartment, which occurs due to the disruption of the blood-nerve barrier and increased permeability of the endoneurial capillaries. The fibrous structure of the endoneurium makes it porous and ineffective as a barrier to the passage of chemicals. As a result, intra-fascicular edema leads to a localized, hypoechoic swelling that can be observed during a ultrasound examination (4). During the chronic phase, Schwann cells are gradually replaced by fibroblasts that produce collagen fibers, and the endoneurial edema is gradually replaced by fibrotic tissue. As a result, the echogenicity of the nerve fascicles may gradually increase, leading to a hyperechoic texture (5). Additionally, dynamic ultrasound enables the evaluation of the median nerve’s mobility or gliding within the carpal tunnel during the post-operative phase (6). This modality is particularly useful for assessing the success of median nerve decompression following a minimally invasive carpal tunnel release procedure (7). By instructing the patient to flex and extend their fingers, clinicians can observe the median nerve’s dynamic movements (7). In postoperative ultrasonography studies of the carpal tunnel, the most commonly analyzed parameters include the median nerve’s cross-sectional area at its widest point along with pre- and post-operative comparative measurements of these values (8).

Although electromyography is still the gold standard for the diagnosis of CTS, B-ultrasonography is being used more and more widely in clinical diagnosis (9, 10). This article reports our experience in diagnosing CTS using B-Mode ultrasound as an additional tool. This included, history and physical examination, electromyography, preoperative B-Mode ultrasound findings, intraoperative direct control, and postoperative findings. To determine the potential role of B ultrasonography in diagnosing CTS.

## Materials and methods

Forty newly diagnosed patients, 12 male and 28 female, average 40.2 aged from 34 to 56 years old, were randomly selected to visit our hospital. Six cases had bilateral symptoms and 34 patients had unilateral symptoms. All patients Complain of numbness of thumb, index finger and middle finger and underwent comprehensive history, physical examination, electrophysiological diagnosis, and B-ultrasonography before operation. Six patients underwent bilateral surgeries for transverse carpal ligament release, and the rest of the patients underwent unilateral surgery. The postoperative follow-up was 10 to 18 months.

Group A was formed by randomly selecting 20 patients from a pool of 34 individuals who were scheduled to undergo unilateral surgery and the diagnosis of CTS was confirmed during surgery. Group B consisted of 20 healthy volunteers randomly selected. The details of Flattening ratio of the median nerve (MN), cross-sectional area of the MN, thickness on the cross-section in both groups are recorded. The details were compared to demonstrate the significance of B-mode ultrasound in diagnosing CTS (Table 1).

**Table 1:** Sonographic findings for two groups.

	Group A	Group B
	(*n* = 20 wrists)	(*n* = 20 wrists)
Flattening ratio of the MN (FR)	2.95 ± 0.42	2.41 ± 0.23
Cross-sectional area of the MN (CSA, cm^2^)	0.15 ± 0.04	0.08 ± 0.02
Thickness on the cross-section of the MN (TCL, mm)	4.45 ± 0.38	3.52 ± 0.51

Group A vs. Group B, *p*-value<0.05. Group A: patients with CTS; Group B: control group.

Criteria for Diagnosis of Carpal Tunnel Syndrome: (1) Complaints of numbness in the thumb, index finger, and middle finger, consistent with median nerve sensory distribution, (2) Physical examination tests are positive, such as Tinel’s sign, Phalen’s maneuver, Durkan’s test, (3) Electrophysiological Tests: Slowing of median nerve sensory and motor conduction velocity across the carpal tunnel, and (4) Ultrasound Imaging: increased cross-sectional area of the median nerve at the proximal carpal tunnel and Swelling of the median nerve or changes in its echotexture.

Exclusion Criteria: (1) Symptoms0020consistent with other conditions such as cervical radiculopathy, peripheral neuropathy, or tenosynovitis, (2) Previous carpal tunnel release surgery on the affected hand, and (3) Severe trauma to the affected wrist or hand.

In this study, we utilized SPSS software (version 27) for data analysis and statistical processing. To assess the differences and correlations between research variables, we employed the *T*-test.

## Instruments and methods

Siemens ACUSON SEQUOIA color ultrasonic diagnostic instrument with linear array probe was used at a frequency of 5–12 MHz. Routine ultrasound examination: The subjects were seated, the upper arm was placed flat on the examination bed, the palm was up, and the wrist joint was slightly extended. The probe was placed vertically on the proximal end of the carpal tunnel. The ulnar lentiform bone, radial scaphoid bone and posterior lunate bone were used as bone markers. The flattening rate of median nerve was calculated (left and right diameters/anteroposterior diameters). Additionally, the cross-sectional area also measured (Measured structures include nerve fascicles, epineurium, and perineurium). Keeping the fingers semi-extended in a neutral position. A high-resolution ultrasound scanner with a linear array transducer, with a frequency of around 13 MHz, is used to measure the CSA at the carpal tunnel inlet.

The surgeon and attending radiologist discussed in detail the B-Mode ultrasound images of each case, combined with relevant clinical data, structural anatomy, and previous radiography, to arrive at the final interpretation of the B-Mode ultrasound imaging study. The median nerve was examined by carpal tunnel using tourniquet under general anesthesia or brachial plexus anesthesia in 40 patients. After the anesthesia takes effect, the patient is laid on the operating table. The upper arm is positioned flat on a support, with the palm facing up and the wrist joint slightly extended. In the surgical area, routine disinfection was performed and sterile drapes were placed. A small incision is made in the palm of the hand near the wrist. The transverse carpal ligament is visualized by carefully dissecting through subcutaneous tissue and palmar fascia. The transverse carpal ligament is carefully cut, releasing the pressure on the median nerve.

Intraoperative findings were observed subjectively and recorded with photos or images and score according to symptoms during postoperative follow-up.

## Results

The history and physical findings of all 40 patients suggested persistent compression median neuropathy. All patients complained of persistent numbness. In 40 cases, symptoms progressed to areas where patients complained of persistent numbness. Other symptoms include pain and weakness. Postoperative symptoms disappeared completely in 35 patients and numbness and tingling were relieved in 5 patients, but there were still varying degrees of pain.

Preoperatively, sonographic imaging was instrumental in diagnosing carpal tunnel syndrome in our patient cohort. B-Mode ultrasound consistently identified abnormalities within the carpal tunnel, such as swelling and deformation of the median nerve or a reduction in the surrounding space, which could indicate the presence of a persistent compression neuropathy. These findings were present in all 40 patients and were found to accurately reflect the intraoperative state of the median nerve. Postoperatively, sonographic images demonstrated a reduction in median nerve swelling and a return to normal carpal tunnel architecture in 35 patients, correlating with the resolution of symptoms. In the remaining five patients, although there was an improvement in nerve morphology, minor persistent changes were observed, aligning with the patients’ residual symptoms.

Surgical exploration and decompression of the carpal tunnel revealed a consistent pattern of median nerve compression among the patients studied. The intraoperative findings confirmed the preoperative sonographic observations, with the transverse carpal ligament contributing to nerve compression in all cases. During surgery, the ligament was released, which was expected to alleviate the pressure on the median nerve. For the majority of patients, this procedure was successful, as evidenced by the resolution of preoperative symptoms. However, in five cases, despite the release, patients experienced ongoing pain post-surgery, suggesting that while the mechanical aspect of nerve compression was addressed, other factors contributing to pain may persist or develop.

**Figure 1:**
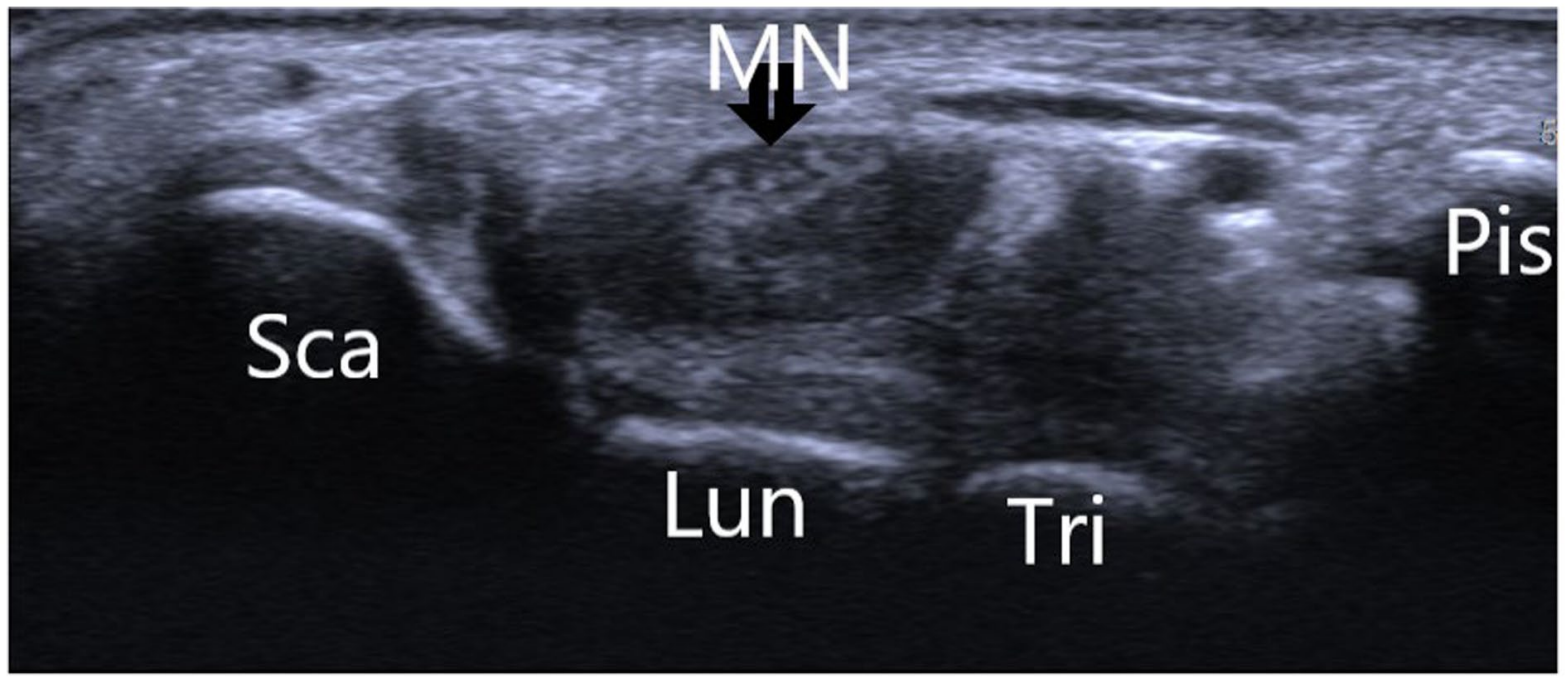
Showing the normal wrist B-ultrasonography image of a 30-year-old asymptomatic male volunteer. *Sca, scaphoid bone; Lun, lunate bone; Tri, triquetral bone; Pis, pisiform bone; MN, median nerve.

**Figure 2:**
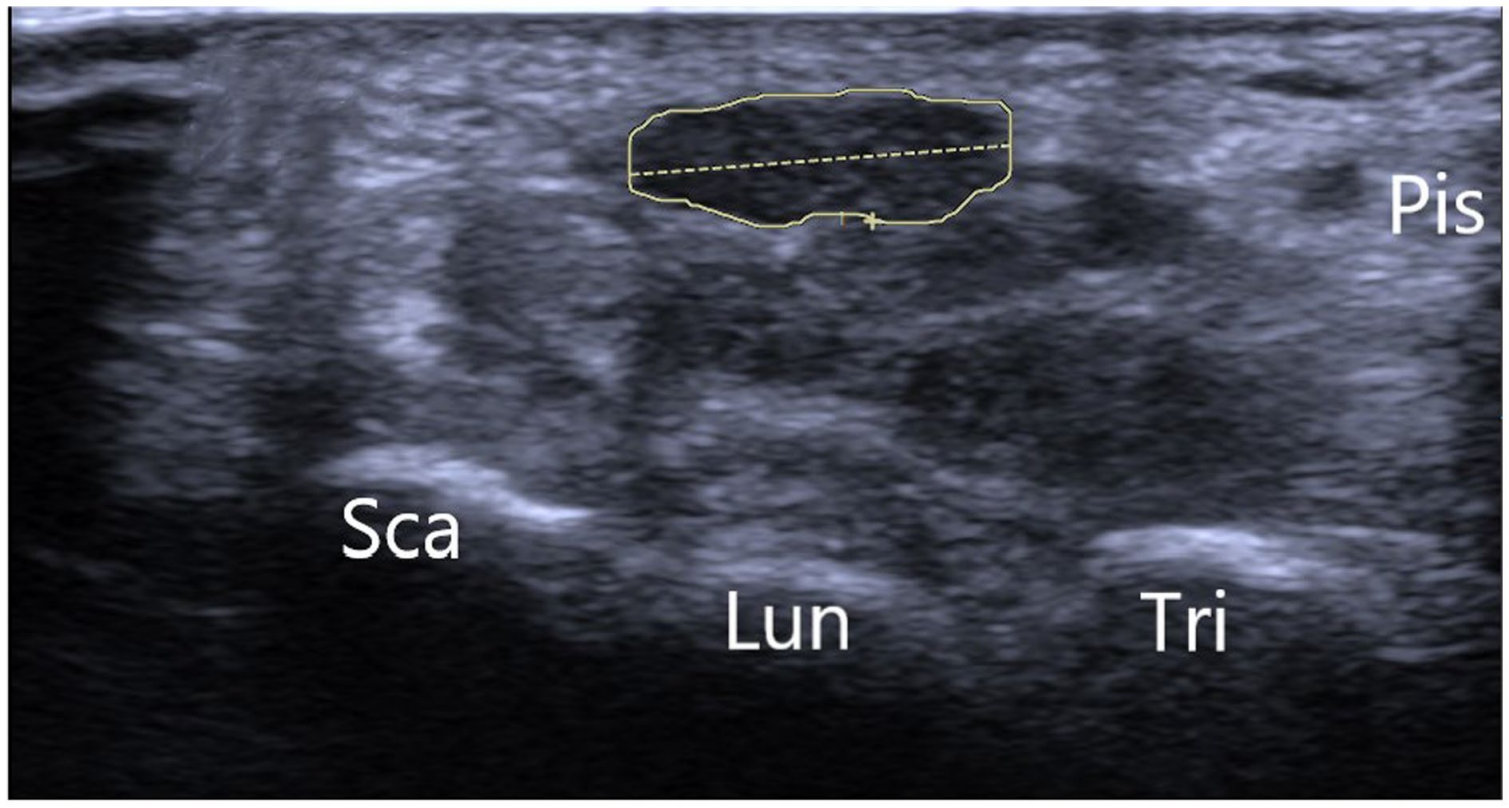
Depicting the B-ultrasonography image of typical carpal tunnel syndrome. *Sca, scaphoid bone; Lun, lunate bone; Tri, triquetral bone; Pis, pisiform bone.

Electrophysiological studies conducted before surgery were consistent with the diagnosis of carpal tunnel syndrome in all patients. Nerve conduction studies and electromyography confirmed the presence of median neuropathy characterized by slowed conduction velocities and altered sensory and motor responses. These findings complemented the clinical presentation of numbness, pain, and weakness in the areas innervated by the median nerve. Postoperative electrophysiological assessments showed significant improvement in nerve conduction, which was in line with the symptomatic relief reported by 35 patients. The remaining five patients showed some electrophysiological improvement, which was not as pronounced, paralleling their partial clinical improvement (Figures 1, 2).

## Discussion

Carpal tunnel syndrome (CTS) is a condition that falls under the category of cumulative trauma disorder (CTDs) and is the most common compression neuropathy of the upper extremities At least 50 percent of cases usually occur in patients between 40 and 60 years of age. Although no research has adequately documented the harmful effects of CTS on the workforce, it is indeed so well known that CTDS are caused by repetitive forceful movements of the upper limbs and, as a result, are relatively common in the workplace (11). Other elderly people are also susceptible to carpal tunnel syndrome after holding grandchildren for long periods of time. Diagnosis of carpal tunnel syndrome depends on history, physical examination, and electrophysiological examination, with electrophysiological examination being the gold standard (12, 13). The application of MR In the diagnosis of carpal tunnel syndrome has also been reported, but it is accompanied by shortcomings such as slow appointment, high cost, and non-real-time measurement (14). With the development of high resolution B-Mode ultrasound, B-Mode ultrasound is more and more accepted by hand surgeons as an auxiliary diagnostic tool for carpal tunnel syndrome. It has the advantages of rapid and non-invasive examination, which can identify the location of nerve entrapment before surgery, and predict the postoperative effect of carpal tunnel release through data analysis (15).

**Figure 3:**
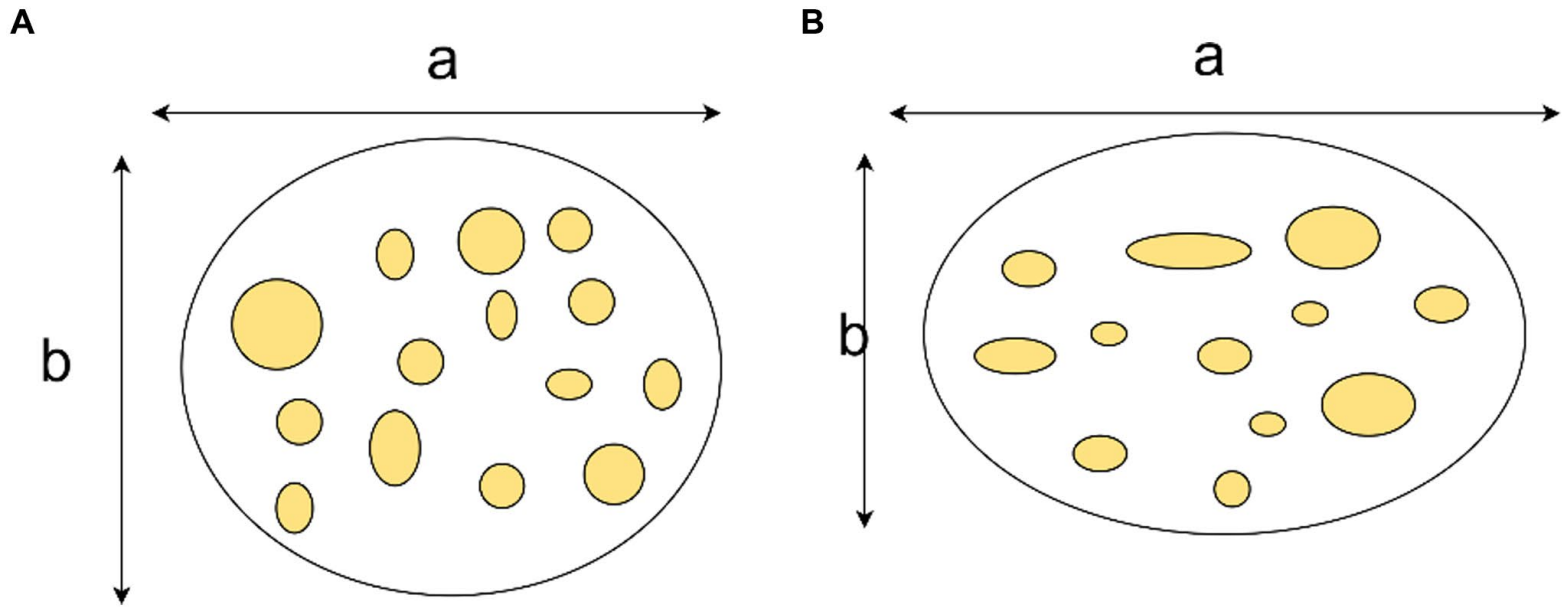
Demonstration of median nerve flattening rate. The median nerve flattening rate equals the ratio of the transverse diameter (a) to the anteroposterior diameter (b). **(A)** Present a normal median nerve. **(B)** Show a median nerve with CTS.

A notable limitation of this study is the small sample size, which may not fully represent the broader population and could limit the generalizability of the findings. Our findings support the utility of preoperative B-Mode ultrasound, which revealed anatomical changes correlating with persistent median nerve symptoms in each of our 40 cases. These changes included a thickened transverse carpal ligament, often exceeding 5 mm, and various manifestations of fascicle edema to the median nerve. Enhancements in nerve echogenicity were also noted, suggesting alterations in nerve characteristics due to compression. Additionally, instances of proximal neural edema exceeding 2 cm were observed. The median nerve flattening rate, measured by comparing the left/right and anteroposterior diameters, provided a precise indicator of compression when showing a statistically significant difference from the healthy side. Moreover, volume changes in the carpal tunnel, particularly at the pisiform and hamate bone levels where the cross-sectional area is less than 0.1 cm^2^, were critical in our assessments. Our standardized imaging techniques and the collaboration between hand surgeons and sonographers have been paramount. For accurate diagnosis, patients should have symptoms lasting more than a month, high-resolution ultrasound equipment must be utilized, and surgeons should engage in detailed discussions with sonographers regarding clinical anatomy (Figure 3).

Images are made by the radiologist to ensure that the entire extent of the carpal tunnel and the transverse ligament are visible. As previously stated, Presazzi A et al. showed a direct relationship between preoperative B-Mode ultrasound and intraoperative findings in 8 patients who did not have carpal tunnel release. We were able to demonstrate a direct relationship between preoperative imaging abnormalities and the scanning and location of intraoperative anatomic compression. We are not saying that ultrasound should replace the standard diagnosis of carpal tunnel syndrome. Ultrasound can also detect abnormal anatomical structures in carpal canal, such as persistent median artery, median nerve bifurcation, variation of motor and palmar cutaneous branches of median nerve, retrograde palmar longus muscle, Martin-Gruber anastomoses, Limburg-Comstock syndrome, etc. (15).

Space-occupying lesions in the carpal canal are also one of the causes of CTS, such as ganglion cysts, fibromas, tenosynovitis and tumors involving the median nerve itself. Depaoli et al. reported two patients with CTS who still had local pain and paresthesia after decompression surgery. Ultrasound examination showed that there was a schwannoma related to the median nerve in the carpal canal, and the patients’ symptoms were relieved after surgical resection (16).

## Conclusion

It can be seen that ultrasonography plays an important role in the etiological diagnosis of CTS. Currently, most sonographers have limited experience in interpreting median nerve compression and neuropathic images of CTS, which also limits the therapeutic techniques of this method. However, we believe that ultrasound can be useful as an adjunct to hand surgeons especially when the physical examination of CTS and the electrodiagnostic evidence is equivocal.

## Data availability statement

The original contributions presented in the study are included in the article/supplementary material, further inquiries can be directed to the corresponding authors.

## Ethics statement

The studies involving humans were approved by Ethical Review Board of Zhejiang Provincial People’s Hospital. The studies were conducted in accordance with the local legislation and institutional requirements. The participants provided their written informed consent to participate in this study.

## Author contributions

QC: Writing – original draft. XZ: Writing – review & editing. YX: Writing – review & editing. YH: Writing – review & editing. CC: Writing – review & editing. PZ: Writing – review & editing.

## References

[1] Crnkovi T Trkulja V Bili R Gapar D Kolundi R. Carpal tunnel and median nerve volume changes after tunnel release in patients with the carpal tunnel syndrome: a magnetic resonance imaging (MRI) study. Int Orthop. (2016) 40:981–7. doi: 10.1007/s00264-015-3052-8, PMID: 26593065

[2] Murphy RX Chernofsky MA Osborne MA Wolson AH. Magnetic resonance imaging in the evaluation of persistent carpal tunnel syndrome. J Hand Surg Am. (1993) 18:113–20. doi: 10.1016/0363-5023(93)90254-Z, PMID: 8423294

[3] Ricci V Ricci C Cocco G Gervasoni F Donati D Farì G. Histopathology and high-resolution ultrasound imaging for peripheral nerve (injuries). J Neurol. (2022) 269:3663–75. doi: 10.1007/s00415-022-10988-1, PMID: 35091803

[4] Härtig F Ross M Dammeier NM Fedtke N Heiling B Axer H. Nerve ultrasound predicts treatment response in chronic inflammatory demyelinating Polyradiculoneuropathy-a prospective follow-up. Neurotherapeutics. (2018) 15:439–51. doi: 10.1007/s13311-018-0609-4, PMID: 29435815 PMC5935640

[5] King R. Microscopic anatomy: normal structure. Handb Clin Neurol. (2013) 115:7–27. doi: 10.1016/B978-0-444-52902-2.00002-3 23931772

[6] Mezian K Ricci V Güvener O Jačisko J Novotny T Kara M. EURO-MUSCULUS/USPRM dynamic ultrasound protocols for wrist and hand. Am J Phys Med Rehabil. (2022) 101:e132–8. doi: 10.1097/PHM.0000000000002005, PMID: 35440527

[7] Chen CH Jaw FS Hu JZ Wu WT Chang KV. Dynamic ultrasound for evaluating the adequacy of median nerve decompression following minimally invasive carpal tunnel release: technical innovation and case study. Heliyon. (2023) 9:e13107. doi: 10.1016/j.heliyon.2023.e13107, PMID: 36711298 PMC9880394

[8] Kapuścińska K Urbanik A. Efficacy of high frequency ultrasound in postoperative evaluation of carpal tunnel syndrome treatment. J Ultrason. (2016) 16:16–24. doi: 10.15557/JoU.2016.0002, PMID: 27103999 PMC4834367

[9] Horng YS Chang HC Lin KE Guo YL Liu DH Wang JD. Accuracy of ultrasonography and magnetic resonance imaging in diagnosing carpal tunnel syndrome using rest and grasp positions of the hands. J Hand Surg Am. (2012) 37:1591–8. doi: 10.1016/j.jhsa.2012.04.040, PMID: 22770417

[10] Deniz F.E. Oksüz E. Sarikaya B.‚ Kurt S. Erkorkmaz U. Ulusoy H. Arslan S. Comparison of the diagnostic utility of electromyography, ultrasonography, computed tomography, and magnetic resonance imaging in idiopathic carpal tunnel syndrome determined by clinical findings. Neurosurgery (2012) 70, 610–616. doi: 10.1227/NEU.0b013e318233868f, PMID: 21869718

[11] Campbell WW. Evaluation and management of peripheral nerve injury. Clin Neurophysiol. (2008) 119:1951–65. doi: 10.1016/j.clinph.2008.03.018 18482862

[12] Vijayavenkataraman S. Nerve guide conduits for peripheral nerve injury repair: a review on design, materials and fabrication methods – science direct. Acta Biomater. (2020) 106:54–69. doi: 10.1016/j.actbio.2020.02.003 32044456

[13] Robinson LR. Traumatic injury to peripheral nerves. J Suppl Clin Neurophysiol. (2004) 57:173–86. 10.1016/s1567-424x(09)70355-1 16124144

[14] Martins RS Siqueira MG Simplício H Agapito D Medeiros M. Magnetic resonance imaging of idiopathic carpal tunnel syndrome: correlation with clinical findings and electrophysiological investigation. Clin Neurol Neurosurg. (2008) 110:38–45. doi: 10.1016/j.clineuro.2007.08.025, PMID: 17920190

[15] Presazzi A Bortolotto C Zacchino M Madonia L Draghi F. Carpal tunnel: Normal anatomy, anatomical variants and ultrasound technique. J Ultrasound. (2011) 14:40–6. doi: 10.1016/j.jus.2011.01.006, PMID: 23396809 PMC3558235

[16] Depaoli R Coscia DR Alessandrino F. Alessandrino, in-continuity neuroma of the median nerve after surgical release for carpal tunnel syndrome: case report. J Ultrasound. (2014) 18:83–5. doi: 10.1007/s40477-014-0127-0 25767645 PMC4353826

